# Supplementary material for: Interdisciplinary perspectives on multimorbidity in Africa: Developing an expanded conceptual model
Source: PLOS Glob Public Health. 2024 Jul 30;4(7):e0003434. doi: 10.1371/journal.pgph.0003434 (PMC11288440; doi:10.1371/journal.pgph.0003434)
Supplement: S1 Checklist — (DOC) [file pgph.0003434.s001.doc]

**Manuscript:** Interdisciplinary perspectives on multimorbidity in Africa: developing an expanded conceptual model

**Consolidated criteria for reporting qualitative studies (COREQ): 32-item checklist**

Developed from:

Tong A, Sainsbury P, Craig J. Consolidated criteria for reporting qualitative research (COREQ): a 32-item checklist for interviews and focus groups. *International Journal for Quality in Health Care*. 2007. Volume 19, Number 6: pp. 349 – 357

| **No. Item** | **Guide questions/description** | **Reported on Page #** |
| --- | --- | --- |
| **Domain 1: Research team and reﬂexivity** |  |  |
| *Personal Characteristics* |  |  |
| 1. Inter viewer/facilitator | Which author/s conducted the interview or focus group? | Page 11 |
| 2. Credentials | What were the researcher’s credentials? E.g. PhD, MD | Pages 10–11 (disciplines specified in more detail in the supplementary material) |
| 3. Occupation | What was their occupation at the time of the study? | Pages 10–11 |
| 4. Gender | Was the researcher male or female? | Implicit through naming the authors on pages 10–11 (gender balance explicitly specified in more detail in the supplementary material) |
| 5. Experience and training | What experience or training did the researcher have? | Beyond the scope of paper to go into this level of detail |
| *Relationship with participants* |  |  |
| 6. Relationship established | Was a relationship established prior to study commencement? | Page 10 |
| 7. Participant knowledge of the interviewer | What did the participants know about the researcher? e.g. personal goals, reasons for doing the research | Beyond the scope of paper to go into this level of detail, but the description of the collaborative research model (9–13) makes clear that the goals and reasons for the research were explicit to all and co-developed further through the research process |
| 8. Interviewer characteristics | What characteristics were reported about the inter viewer/facilitator? e.g. Bias, assumptions, reasons and interests in the research topic | Pages 9, 10, 27–28 |

| **Domain 2: study design** |  |  |
| --- | --- | --- |
| *Theoretical framework* |  |  |
| 9. Methodological orientation and Theory | What methodological orientation was stated to underpin the study? e.g. grounded theory, discourse analysis, ethnography, phenomenology, content analysis | Pages 9, 11–12 |
| *Participant selection* |  |  |
| 10. Sampling | How were participants selected? e.g. purposive, convenience, consecutive, snowball | Page 10 |
| 11. Method of approach | How were participants approached? e.g. face-to-face, telephone, mail, email | Page 10 |
| 12. Sample size | How many participants were in the study? | Page 10 |
| 13. Non-participation | How many people refused to participate or dropped out? Reasons? | Beyond the scope |
| *Setting* |  |  |
| 14. Setting of data collection | Where was the data collected? e.g. home, clinic, workplace | Page 9 |
| 15. Presence of non-participants | Was anyone else present besides the participants and researchers? | Beyond the scope |
| 16. Description of sample | What are the important characteristics of the sample? e.g. demographic data, date | Page 10, figure 1, supplementary material |
| *Data collection* |  |  |
| 17. Interview guide | Were questions, prompts, guides provided by the authors? Was it pilot tested? | Page 11 – there reader is guided to the more detailed workshop proceedings |
| 18. Repeat interviews | Were repeat inter views carried out? If yes, how many? | N/A |
| 19. Audio/visual recording | Did the research use audio or visual recording to collect the data? | N/A |
| 20. Field notes | Were ﬁeld notes made during and/or after the inter view or focus group? | Page 12 |
| 21. Duration | What was the duration of the interviews or focus group? | Beyond the scope |
| 22. Data saturation | Was data saturation discussed? | Beyond the scope |
| 23. Transcripts returned | Were transcripts returned to participants for comment and/or correction? | Page 12 |
| **Domain 3: analysis and ﬁndings** |  |  |
| *Data analysis* |  |  |
| 24. Number of data coders | How many data coders coded the data? | Page 12 |
| 25. Description of the coding tree | Did authors provide a description of the coding tree? | Page 12 and 14 |
| 26. Derivation of themes | Were themes identiﬁed in advance or derived from the data? | Page 12 |
| 27. Software | What software, if applicable, was used to manage the data? | Google docs (open coding) |
| 28. Participant checking | Did participants provide feedback on the ﬁndings? | Pages 12–13 |
| *Reporting* |  |  |
| 29. Quotations presented | Were participant quotations presented to illustrate the themes/ﬁndings? Was each quotation identiﬁed? e.g. participant number | Page 16, 19, 21 – not feasible to present individual identifiers but session / group number is named where applicable |
| 30. Data and ﬁndings consistent | Was there consistency between the data presented and the ﬁndings? | Page 13–21 |
| 31. Clarity of major themes | Were major themes clearly presented in the ﬁndings? | Page 13–21 |
| 32. Clarity of minor themes | Is there a description of diverse cases or discussion of minor themes? | Page 15–27 |
