# Supplementary material for: Interdisciplinary perspectives on multimorbidity in Africa: Developing an expanded conceptual model
Source: PLOS Glob Public Health. 2024 Jul 30;4(7):e0003434. doi: 10.1371/journal.pgph.0003434 (PMC11288440; doi:10.1371/journal.pgph.0003434)
Supplement: S1 Table — Details measures taken during the research to promote equitable partnership within this collaboration. (DOCX) [file pgph.0003434.s002.docx]

**S1 Table. Reflexivity statement***

| **Element** | **Approaches** |
| --- | --- |
| **Workshop conceptualization** | 1. **How does this research address local research and policy priorities?**   Multimorbidity is an increasing concern globally. Current understandings of multimorbidity mainly reflect research and perspectives from high-income settings. This research contributes to the development of a sub-Saharan African specific understanding of and agenda for responding to multimorbidity. |
|  | 1. **How were local researchers involved in research design?**   The workshop organising committee included researchers from countries in sub-Saharan Africa, and the workshop was co-designed with invited speakers, most of whom live and work within the region. The workshop was designed to be open ended, a starting point for bringing researchers from sub-Saharan Africa together to discuss opportunities for further collaboration. All contributors were involved in these discussions. |
| **Workshop management** | 1. **How has funding been used to support the local research team(s)?**   Through research funding from the United Kingdom, we made bursaries available for those who could otherwise not travel to the event. This includes African early-career researchers (PhD students). |
| **Data acquisition and analysis** | 1. **How are research staff who conducted data collection acknowledged?**   Data collection in this context is a synthesis of ongoing research activity and contextualized knowledge that all participants contributed to producing. All contributors have been acknowledged as authors. |
|  | 1. **How have members of the research partnership been provided with access to study data?**   All formal conversations had a rapporteur to ensure insights were captured. All contributors to the workshop have a full account of the discussion and conclusions. |
|  | 1. **How were data used to develop analytical skills within the partnership?**   The workshop was designed to facilitate and strengthen critical thinking and analysis across disciplinary and disease-specific knowledge bases. |
| **Data interpretation** | 1. **How have research partners collaborated in interpreting study data?**   The work followed a semi-structured approach based on conceptual spheres identified in advance of the workshop. Partners were asked to contribute to any specific conversation they felt they could. Working groups were subsequently formed to take forward major themes emerging from workshop and formulate into papers. All participants have been offered the opportunity to join groups that fit their domains of expertise. |
| **Drafting and revising for intellectual content** | 1. **How were research partners supported to develop writing skills?**   Detailed notes of proceedings were taken by a team of rapporteurs made up of early-career researchers, mentored by more senior colleagues. This mentoring approach was continued into the writing of workshop proceedings and other outputs. |
|  | 1. **How will research products be shared to address local needs?**   The synthesis of opinion and current state of the art was contextualized throughout its production by the presence of local research and policy leaders. Published proceedings will be made available open access, as will any further outputs resulting from the workshop. We will use our network to distribute outputs. |
| **Authorship** | 1. **How is the leadership, contribution, and ownership of this work by LMIC researchers recognised within the authorship?**   All author and leader contributions to the workshop and resulting outputs have been acknowledged in line with  CRediT contributor roles taxonomy. |
|  | 1. **How have early career researchers across the partnership been included within the authorship team?**   We identified early career researchers within our network who were financially supported to attend the workshop, and who have been mentored through the writing process, as above. |
|  | 1. **How has gender balance been addressed within the authorship?**   We have not specifically addressed gender balance in terms of selection of invitation to take part. We will report the aggregate group composition by sex, for transparency. |
| **Training** | 1. **How has the project contributed to training of LMIC researchers?**   LMIC researchers within the group form key leaders in the area. The overall aims of the workshop and its outputs are to ensure these efforts and results are recognized, and we aimed for key roles in chairing and leading discussions to represent all researchers present. |
| **Infrastructure** | 1. **How has the project contributed to improvements in local infrastructure?**   There are no tangible changes to local infrastructure given the timeframe and objectives of the work. |
| **Governance** | 1. **What safeguarding procedures were used to protect local study participants and researchers?**   The workshop did not require formal ethical review. The host organisation, Malawi-Liverpool-Wellcome Trust Clinical Research Programme has safeguarding mechanisms, including anonymous and other reporting procedures. These were shared with the group from the outset of the collective work. |

*This statement has been prepared in line with recent consensus recommendations described in: Morton B, Vercueil A, Masekela R, et al. Consensus statement on measures to promote equitable authorship in the publication of research from international partnerships. *Anaesthesia* 2022; 77: 243–7.
