# Supplementary material for: Interdisciplinary perspectives on multimorbidity in Africa: Developing an expanded conceptual model
Source: PLOS Glob Public Health. 2024 Jul 30;4(7):e0003434. doi: 10.1371/journal.pgph.0003434 (PMC11288440; doi:10.1371/journal.pgph.0003434)
Supplement: S2 Table — Provides an overview of gender balance, geographical representation, and career stage within the collaboration. (DOCX) [file pgph.0003434.s003.docx]

**S2 Table. Workshop Participant Summary Table**

| **Indicator** | **Value (n=60)** |
| --- | --- |
| Gender balance (n, % female) | 32 (53%) |
| LMIC inclusion (n, % LMIC-based researchers) | 41 (68%) |
| Career stage (n, % early-career) | 35 (58%) |
