# Supplementary material for: Interdisciplinary perspectives on multimorbidity in Africa: Developing an expanded conceptual model
Source: PLOS Glob Public Health. 2024 Jul 30;4(7):e0003434. doi: 10.1371/journal.pgph.0003434 (PMC11288440; doi:10.1371/journal.pgph.0003434)
Supplement: S3 Table — Describes each participant’s institution(s) and disciplinary and regional expertise. (DOCX) [file pgph.0003434.s004.docx]

**S3 Table. Full participant breakdown**

| **Name** | **Institution** | **Discipline / field** | **Regional expertise in sub-Saharan Africa** |
| --- | --- | --- | --- |
| Gift Treighcy Banda | Malawi-Liverpool-Wellcome Trust Clinical Research Programme, Malawi  Liverpool School of Tropical Medicine, UK | Social science, health systems | Malawi |
| Fanuel Bickton | Malawi-Liverpool-Wellcome Trust Clinical Research Programme, Malawi  The Kamuzu University of Health Sciences, Malawi | Cardiorespiratory physiotherapy | Malawi |
| Abbi-Monique Bilungula Mamani | University of Kinshasa, Democratic Republic of Congo | Pulmonary rehabilitation | Democratic Republic of Congo |
| Edna Bosire | Brain and Mind Institute, Aga Khan University, Kenya  University of The Witwatersrand, South Africa | Medical anthropology | Kenya, South Africa, Malawi |
| Christopher Bunn | Malawi Epidemiology and Intervention Research Unit, Malawi  University of Glasgow, UK | Sociology | Malawi |
| Claire Calderwood | Biomedical Research and Training Institute, Zimbabwe  London School of Hygiene & Tropical Medicine, UK | Respiratory medicine | Zimbabwe |
| Clare Chandler | London School of Hygiene & Tropical Medicine, UK | Medical anthropology | Zimbabwe, Malawi, Tanzania, Uganda |
| Marlen Stacy Chawani | Malawi-Liverpool-Wellcome Trust Clinical Research Programme, Malawi  Health Economics and Policy Unit, Malawi | Health information systems | Malawi |
| Edith Chikumbu | Malawi Epidemiology and Intervention Research Unit, Malawi | Social science, medical doctor | Malawi |
| Beatrice Chinoko | Malawi-Liverpool-Wellcome Trust Clinical Research Programme, Malawi | Nursing, community medicine | Malawi |
| Mphatso Chisala | Malawi Epidemiology and Intervention Research Unit, Malawi | Medical doctor | Malawi |
| Jonathan Chiwanda | Malawi Ministry of Health, Malawi | Policymaker | Malawi |
| Mia Crampin | Malawi Epidemiology and Intervention Research Unit, Malawi  University of Glasgow, UK  London School of Hygiene & Tropical Medicine, UK | Clinical epidemiology | Malawi |
| Justin Dixon | Biomedical Research and Training Institute, Zimbabwe  London School of Hygiene & Tropical Medicine, UK | Medical anthropology | Zimbabwe, South Africa |
| Sarah Drew | University of Bristol, UK | Social science, musculoskeletal research | Zimbabwe, The Gambia, South Africa |
| Lindsay Farrant | University of Cape Town, South Africa | Palliative care medicine | South Africa |
| Rashida Ferrand | Biomedical Research and Training Institute, Zimbabwe  London School of Hygiene & Tropical Medicine, UK | Clinical epidemiology, HIV and sexual and reproductive health | Zimbabwe |
| Mtisunge Gondwe | Malawi-Liverpool-Wellcome Trust Clinical Research Programme, Malawi  Liverpool School of Tropical Medicine, UK | Nursing, health systems, bioethics | Malawi |
| Celia Gregson | University of Bristol, UK | Geriatric medicine | Zimbabwe, The Gambia, South Africa |
| Richard Harding | King’s College London, UK | Palliative care | Ghana, Malawi, Zimbabwe |
| John Hurst | University College London, UK | Respiratory medicine | Uganda |
| Dan Kajungu | Makerere University, Uganda | Biostatistics | Uganda |
| Stephen Kasenda | Malawi Epidemiology and Intervention Research Unit, Malawi | Medical doctor | Malawi |
| Winceslaus Katagira | Makerere University Lung Institute, Uganda | Medical doctor, lung health | Uganda |
| Andre-Pascal Kengne | South African Medical Research Council, South Africa | Medical doctor and internist, non-communicable diseases | South Africa |
| Duncan Kwaitana | The Kamuzu University of Health Sciences, Malawi | Palliative care | Malawi |
| Naomi Levitt | University of Cape Town, South Africa | Endocrinology, epidemiology | South Africa |
| Felix Limbani | Malawi-Liverpool-Wellcome Trust Clinical Research Programme, Malawi | Social science, health systems | Malawi |
| Emily Mendenhall | Georgetown University, US | Medical anthropology | Kenya, South Africa |
| Adwoa Bemah Boamah Mensah | Kwame Nkrumah University of Science and Technology, Ghana | Nursing, oncology | Ghana |
| Modai Mnenula | University of Malawi, Malawi | Family medicine | Malawi |
| Ben Morton | Liverpool School of Tropical Medicine, UK | Critical care | Malawi |
| Mosa Moshabela | University of KwaZulu-Natal, South Africa | Family medicine, public health | South Africa |
| Lovemore Mupaza | Midlands State University, Zimbabwe | Development practitioner | Zimbabwe |
| Maud Mwakasungula | Malawi NCD Alliance, Malawi | Non-communicable diseases advocacy | Malawi |
| Wisdom Nakanga | Malawi Epidemiology and Intervention Research Unit, Malawi  University of Exeter, UK | Medical doctor, non-communicable diseases | Malawi |
| Chiratidzo Ndhlovu | University of Zimbabwe, Zimbabwe | Nephrology, essential medicines policy | Zimbabwe |
| Misheck Julian Nkhata | Teeside University, UK | Medical anthropology | Malawi |
| Kennedy Nkhoma | King’s College London, UK | Nursing, health services research, palliative care | Malawi |
| Owen Nkoka | Malawi Epidemiology and Intervention Research Unit, Malawi  University of Glasgow, UK | Epidemiology | Malawi |
| Edwina Addo Opare-Lokko | University of Ghana Medical School, Ghana | Family medicine | Ghana |
| Mayowa Owolabi | University of Ibadan, Nigeria | Neurology | Nigeria |
| Nasheeta Peer | South African Medical Research Council, South Africa | Cardiovascular diseases and diabetes epidemiology, health systems | South Africa |
| Nozgechi Phiri | Malawi Epidemiology and Intervention Research Unit, Malawi | Social science | Malawi |
| Jacob Phulsa | Malawi-Liverpool-Wellcome Trust Clinical Research Programme, Malawi | Public health | Malawi |
| Alison Price | Malawi Epidemiology and Intervention Research Unit, Malawi  London School of Hygiene & Tropical Medicine, UK | Epidemiology | Malawi |
| Jamie Rylance | Malawi-Liverpool-Wellcome Trust Clinical Research Programme, Malawi  Liverpool School of Tropical Medicine, UK | Respiratory medicine, critical care | Malawi |
| Charity Salima | Achikondi Women and Community Friendly Health Services, Malawi | Nursing, community medicine | Malawi |
| Sangwani Salimu | Malawi-Liverpool-Wellcome Trust Clinical Research Programme, Malawi  Liverpool School of Tropical Medicine, UK | Social science, public health | Malawi |
| Alan Silman | University of Oxford, UK | Rheumatology, epidemiology | General LMIC interest |
| Ibrahim Gibunje Simiyu | Liverpool School of Tropical Medicine, UK | Medical doctor, public health, health systems | Tanzania, Malawi |
| Sally Singh | University of Leicester, UK | Pulmonary and cardiac rehabilitation | Uganda, Malawi |
| Stephen Spencer | Liverpool School of Tropical Medicine, UK | Critical care | Malawi |
| Joachim Sturmberg | University of Newcastle, Australia | General medicine, complexity science | - |
| Tsaone Tamuhla | University of Cape Town and South African National Bioinformatics Institute, University of the Western Cape, South Africa | Bioinformatics | South Africa |
| Mandikudza Tembo | Biomedical Research and Training Institute, Zimbabwe  London School of Hygiene & Tropical Medicine, UK | Adolescent health, sexual and reproductive health | Zimbabwe |
| Nicki Tiffin | South African National Bioinformatics Institute, University of the Western Cape, South Africa | Genomics, epidemiology, health informatics | South Africa |
| Myrna Van Pinxteren | University of Cape Town, South Africa | Medical anthropology, public health | South Africa |
| Elizabeth Vale | University of the Witwatersrand, South Africa | Medical anthropology | South Africa |
| Nateiya Mmeta Yongolo | Liverpool School of Tropical Medicine, UK | Medical doctor, health economics | Tanzania, Malawi |
