# Supplementary material for: Interdisciplinary perspectives on multimorbidity in Africa: Developing an expanded conceptual model
Source: PLOS Glob Public Health. 2024 Jul 30;4(7):e0003434. doi: 10.1371/journal.pgph.0003434 (PMC11288440; doi:10.1371/journal.pgph.0003434)
Supplement: Attachment — Submitted filename: Response letter.pdf [file pgph.0003434.s005.pdf]

17th October 2023

Dear Editors of PLOS Global Public Health,

Re: Re: Interdisciplinary perspectives on multimorbidity in Africa: developing an expanded conceptual model

Thank you for considering our article for publication in PLOS Global Public Health. The paper was rejected by an academic editor for reasons that include data availability, clarity of research question, and reporting of the analysis. We have decided to revise our submission based on the feedback provided and resubmit. Below we detail the reasons for rejection provided, and how we have responded.

We hope that our responses are satisfactory and that our article can be reconsidered.

Yours faithfully,

Justin Dixon, on behalf of the authors

Academic editor

Comment 1. "No defined objective or research question is made available in the article".

> ***Response 1.*** Thank you for this comment. We believed that we have clearly articulated the aim of the research, however have revised the abstract and introduction to more clearly articulate the question underlying the aim. The abstract lines 126-129 now reads:
>
> > *"This article presents the findings from an interdisciplinary research initiative that drew together 60 academic and applied partners working in 10 African countries to answer the question: how useful is the concept of multimorbidity within Africa? Can the concept of multimorbidity be adapted to context to optimise its transformative potentials?"*
>
> The introduction **lines 196-200** now read:
>
> > *"Responding to this need, this article presents the findings and outcomes from an interdisciplinary research initiative to interrogate the conceptual underpinnings of multimorbidity research and care in Africa. It sought to address the questions: how useful is the concept of multimorbidity within the African context? Can the concept be adapted for it to be more context-sensitive, cross-cutting, and more transformative?"*

Comment 2. "The method described is information collected from a 3 days workshop and its notes. The major data are collected through detailed notes of proceedings taken by a team of rapporteurs made up of early-career researchers, mentored by more senior colleagues. . These notes and the other source documents are not submitted".

> **Response 2.** Thank you. We originally did not supply the original source materials for this manuscript for ethical reasons, specifically to protect the identities of participants. However, we have now made the de-identified source materials available through Harvard Dataverse, and have amended the data availability statement accordingly.

Comment 3. "The details of data analysis is not available and the authors of the opinion that there is no distinction between the data collection and data analysis-- So there are some gaps related to the data analysis also".

> ***Response 3.*** Thank you for this comment. We have amended the analysis section to provide greater detail about the analysis process. We have further clarified what we meant when we

argued that there is a fine line between data collection and analysis, which we strongly contend is blurred in the context of strong co-productive research. However, we have distinguished the kind of analysis that occurred during the process of data collection, and the *formal* analysis procedures. undertaken afterwards. The changes are made **lines 279-299** now read:

> *"As this was an iterative, co-productive process, (21) analysis began during the workshop itself, with all participants in engaging in critical reflection during sessions and collaboratively drawing out major cross-cutting issues in the final session.(22) Source material for formal analysis following the workshop included detailed notes of proceedings, including both focus groups and plenary discussions, taken by the rapporteur team. Where necessary, the rapporteurs went back to individuals for clarification, which were absorbed into their notes; all notes were collated and reconciled to produce a unified account of both focus-group and plenary discussions made available to all. Also included for analysis were the flip chart pages composed during focus groups and the Microsoft Word documents produced using a shared screen during plenary discussion. The joint first authors (JD, BM, MJN, AS, IGS, SS, MVP) subsequently conducted iterative inductive thematic analysis using a team-based, 'open coding' approach that is designed to optimise inter-coder consensus in the context of collaborative research (27) Source documents were uploaded onto a shared drive, which enabled the coding team to collaboratively identify initial themes emerging from the data. A series of analysis meetings were used to consolidate these codes into an initial coding framework, while working towards codes progressively higher orders of abstraction to group and explain lower-level themes. The final code tree, which we present in the results section, was then taken to the larger working group and used to develop of an expanded model of multimorbidity. Developing and refining this model was itself an iterative process, involving several analysis meetings discussing subsequent iterations, which was then shared with the wider collaborator group along with the draft manuscript for further comment, discussion, and refinement".*
