# Supplementary material for: Interdisciplinary perspectives on multimorbidity in Africa: Developing an expanded conceptual model
Source: PLOS Glob Public Health. 2024 Jul 30;4(7):e0003434. doi: 10.1371/journal.pgph.0003434 (PMC11288440; doi:10.1371/journal.pgph.0003434)
Supplement: Attachment — Submitted filename: PLOS-GPH_Response_Letter_24_05_11.docx [file pgph.0003434.s007.docx]

Dear Editors of PLOS Global Public Heath,

**Re: Interdisciplinary perspectives on multimorbidity in Africa: developing an expanded**

**conceptual model**

We would like to express our gratitude for the insightful and constructive reviewer comments on our paper, “Interdisciplinary perspectives on multimorbidity in Africa: developing an expanded conceptual model”. We have revised our manuscript accordingly and believe that the paper is much strengthened as a result. We hereby resubmit with highlights to mark alterations. Following the reviewers’ comments, the main changes have been made to expand the methodology and to reduce the overall length of the manuscript, with other revisions throughout. In addition to the reviewers’ comments, we have made minor edits to ensure that the document is consistent with the changes made. Below, we respond to each of the reviewers’ points in turn.

We hope that you will agree that the paper is now strengthened and ready for publication.

Yours faithfully,

Justin Dixon and Felix Limbani, on behalf of the authors

| **Reviewer comment** | **Authors’ response** |
| --- | --- |
| **Reviewer: 1** |  |
| The manuscript is worthy of publication in PLOS ONE because the authors write about important issues for the future of global health. The methods as described are sufficiently rigorous, and the data from the workshop supports the authors’ conclusions. However, the manuscript could be strengthened with revisions. As articulated below, I suggest the authors focus on revising the text to clarify and condense the text as well as describe the research methods in greater detail. | We thank the reviewer for their favourable view of the paper and for the constructive feedback on how we can further strengthen the manuscript. |
| **Major Feedback** |  |
| Results section:  a. Needs to include more data on what is unique about the African context (e.g., more single disease-donor funded health care, higher background rates of poverty and higher potential for catastrophic costs compared to high income countries, higher background levels of infectious diseases) in all three thematic areas to answer the research questions. | Thank you for this valuable suggestion. We believe that the reviewer’s suggestion further down to include a more explicit statement in the introduction about what makes multimorbidity unique in Africa presents opportunity to also address this comment. We have added not just a statement but an expanded paragraph describing published literature around that makes the multimorbidity challenge unique in the African settings. This sets the background for the findings which are more concentrated on what the uniqueness of multimorbidity in Africa means for how we conceptualize multimorbidity (rather than getting too tied down with describing the health systems and epidemiological context across every theme, which is not the research question). The introduction (p. 7-8) now reads:  “With multimorbidity a growing research priority in Africa, the challenges it presents in this setting is becoming clearer. Like many intractable health system challenges, multimorbidity can be linked to centuries of colonialisation and racial discrimination that have systematically limited access to educational opportunities, employment, adequate housing, and basic healthcare.(7,8) Chronic infectious diseases including HIV and tuberculosis (TB) that have thrived in such contexts increasingly intersect with non-communicable diseases (NCDs) including diabetes, cardiovascular diseases, and mental illness which have sharply risen due to the expansion of HIV treatment coverage, rising life expectancies, and lifestyles of poverty and urbanisation.(7,9,10) The resulting multimorbidity burden has been characterised in terms of the ‘colliding epidemics’ of chronic infectious and NCDs, generally affecting a younger, more economically-active demographic than in HICs, socially patterned along the lines of intersectional inequalities.(4,11) Most people living with multimorbidity (PLWMM) rely on public sector care, which generally remains donor-dependent, siloed, and heavily biased towards HIV and TB, with very limited funding available for NCDs.(4,7) There remains a stark disparity in quality of care for people with NCDs and, despite ongoing efforts, limited progress with integrating NCD care with programmes chronic infectious diseases.(12–14) Fragmented, unevenly-resourced systems place a heavy health, social, and economic on burden on PLWMM, families, and wider social networks, undermining abilities to self-manage conditions and heightening the risk of complications and further morbidities.(15,16). "  In light of these additions to the introduction, we have made corresponding minor changes across the results section to ensure consistency in wording, to expand on points made in the introduction, and, where necessary, to reiterate/expand on what makes MM unique in African settings (e.g. colliding HIV-NCD epidemic, verticalized care, strong role of family and context of medical pluralism). Thank you for assisting us to make this clearer across the article. |
| b. Some sentences seem to be in the wrong paragraphs for their thematic focus and should be rearranged. (e.g., sentences at line 338- 351 can go in theme two; sentences at line 377- 380 should move to theme one) | Thank you for pointing this out. The sentence at 338-351 has been deleted, as similar information is already contained in theme 2. The passage on pages 15-16 now reads:  Disease-centred thinking is, as one family physician argued, so entrenched within current systems that it is extremely challenging to step beyond this frame of reference:  “The challenge is that the system we work in does not accommodate a transformation in thinking; it keeps bringing us back to a focus on diseases” (Session 1, plenary, rapporteur notes)”.  We would contend the value of keeping line 377-380 where it is, because we are making the specific point that what matters to patients is not necessarily whether their disease is communicable or non-communicable, but the effects that it has on their symptoms and quality of life. Thus, it fits within this section (and it has now been deleted from theme 1 meaning there is no repetition). |
| c. Themes could be named more clearly communicate the focus-- in plain language if possible. For example, theme one may be renamed “inability for current definition to address deficits in care for people living with MM (PLWMM) in Africa”. Theme two may be renamed “potential for revised definitions to centre the experience of living with MM in Africa”. Theme three may be renamed “need for revised definitions to respond to varied conceptual priorities amongst stakeholders”. | Thank you, we have revised the theme names, drawing on the reviewer’s suggestions (which have been further edited by the author group).  Theme 1: Multimorbidity is currently centred on constituent diseases  Theme 2: Potential for revised concepts to centre the priorities, needs, and social context of PLWMM  Theme 3: Need for revised concepts to respond to varied conceptual priorities amongst stakeholders |
| d. More sentences in the results section should start with words like “participants stated, clinicians reported, community representatives emphasized, researchers elaborated”. The way it is currently written (with fewer sentences starting this way) makes many sentences in the results section sound like definitive facts rather than the thoughts of the participants. This is especially problematic in theme two as participants are speaking on behalf of PLWMM. | Thank you for the suggestions on how to make clearer the perspectives from which our findings have been drawn (rather than overly factual). We have added numerous phrases to this effect through the results, especially in the second theme. E.g. “participants emphasised…” (p.18), and “…some participants including community advocates argued that” (p. 18), and “Challenges frequently observed in qualitative research included difficulties…” (p. 20) |
| e. The results section would be much more readable, and the credibility of the analysis would be enhanced by moving quotes from boxes into the text to support the relevant statements. | Thank you, we have now included the quote in text throughout |
| 3) The text could be condensed and summarized throughout. The discussion and analytic framework sections are particularly lengthy and could be shortened. a. The authors do not need to review each thematic area separately in the discussion section. I suggest the authors pull out the main overarching points, which I have interpreted to be:  i) The data in this study supports the conclusions of other authors who have stated that the concept of MM must move away from the biomedical tendency to define people by their disease.  ii) Participants in this study stated that there has been a great deal of person-centred theorizing about the concept of MM, but African health systems have not responded to this literature by providing more holistic care. For example, our results are aligned with other African qualitative researchers who have pointed out that health systems must address patients’ socioeconomic needs to prevent them from tipping into catastrophic consequences.  iii) As seen in our proposed definition, we believe MM and should incorporate concepts from systems, complexity, Ubuntu, and syndemic theories to demonstrate the need for holistic, integrated models of care for PLWMM in Africa. To that end, we have incorporated these theories with workshop data to develop a model depicting intertwined biological, socio-cultural, political, economic, and environmental factors. | We agree with the reviewer and have condensed the text throughout. In particular, the reviewer’s suggestion for pulling out the main overarching points in the discussion was very helpful, and we have edited the discussion section in line with their proposal for bringing out the three key points, while recognizing there are some additional subtleties to the argument that we feel important to retain (e.g. around decolonizing multimorbidity, and the need to foreground common consequences of MM). The discussion (up to the presentation of the expanded model) now reads on pages 23-25:  Multimorbidity presents a major health system challenge for African countries. We identified three core themes that are pertinent to the conceptualisation of multimorbidity in Africa: the disease-centricity of current concepts; the potential for revised concepts to foreground what matters to PLWMM; and the need to accommodate varied conceptual priorities amongst different stakeholders. Building on this framework, in the following, we place these core themes into conversation with current global health scholarship on multimorbidity, thereby developing an expanded model of multimorbidity that can underpin a coordinated but context-specific response to multimorbidity in Africa.  Our analysis supports the conclusions of others who have argued that the current definition of multimorbidity as two-or-more-long-term conditions promotes a static, additive rendering of disease that treats illness as the sum of its parts and thus continues to define people by their diseases.(19,29,30) One consequence of this disease-centred framing, as Blarikom et al.(19) have argued, is that many researchers working within the field have become preoccupied and indeed, largely paralysed by the search for standard biomedical definition, which can ultimately undermine the concept’s anticipated potential of moving us towards more person-centred research and care.(19,29,30) Recent calls to “decolonise”(31) multimorbidity highlight the need to decentre NCDs associated with ageing to recognise the ‘colliding epidemics’ of communicable and NCDs that characterise multimorbidity in many low-resource settings.(4) While this may have important implications for integrating care across historically separated disease domains, the workshop proceedings suggest that decolonising multimorbidity means more than adjusting its constituent diseases. Rather, it means a more decisive shift away from models of research and care that define and categorise people by their diseases.  Such a shift, our analysis shows, requires foregrounding the priorities, needs, and social context of PLWMM. Calls for more holistic approaches resound in literature and align with theoretical perspectives from multiple disciplines. This includes eco-social theory from epidemiology;(32) the syndemic framework,(7,33) the theory of recursive cascades(34) and burden of treatment theory(16) from the social sciences, and novel applications of complexity theory to multimorbidity within the primary care sciences.(29) Despite this, attempts to integrate care have not proven to be especially ‘person-centred’ in practice. Current models, such as South Africa’s Integrated Chronic Disease Management (ICDM) programme, have often been built based on vertical disease programmes and struggled to overcome legacies of system fragmentation, uneven resourcing of conditions, and individualized responsibility for self-management.(13) A growing number of qualitative studies reporting PLWMM’s perspectives from Malawi,(16) Ghana(35), Ethiopia,(36) and South Africa(37) show that PLWMM are unable to take responsibility in self-management if they experience a ‘lack’ of health services, information, and basic necessities. Such lack in turn feeds into a cycle of precariousness that negatively impacts people’s ability to cope, often sending them down a slippery slope – or in complexity theory terms, over ‘tipping points’(29) – towards further disability and decline. Also appealed to but rarely prioritised in practice is the active involvement of caregivers and support networks in the provision of care for PLWMM.(13,38) Here, African social network theories such as Ubuntu – promoting mutual caring through compassion, reciprocity, dignity, and humanity – could be a useful concept, as they potentially cultivate resilience by creating a shared identity between PLWMM and carers, allowing them to flourish even when living in precarity.(38) The delivery of such care could involve the decentralisation of chronic care through health and social care workers trained in holistic, community-based care models – as elaborated, for instance, within the syndemic care framework.(7,39)  Our findings suggest that a focus on common consequences of multimorbidity holds promise for bringing together different conceptual priorities of stakeholders. If, indeed, many of the needs and treatment burdens experienced by PLWMM are often not disease specific, such a disease-agnostic focus could bring together disciplines and specialities in a way that has eluded the abstract and seemingly irresolvable attempts to pin down multimorbidity and its causal pathways in pathophysiological terms. The broader point is that the distinction between causes and consequences itself begins to break down once we recentre the circumstances and trajectories of PLWMM, from whose perspective ‘consequences’ of a living with multimorbidity today may be ‘causes’ of further problems tomorrow.(34) By centring PLWMM and their ability to lead healthy, fulfilling and independent lives, we may better align medical and lay models of multimorbidity, such that it may not only be more cross-cutting and useful but also, perhaps, carry fewer negative connotations as a label. Figure 2 presents an expanded model of multimorbidity that folds in the findings of our analyses. |
| **Minor Feedback** |  |
| Introduction  • Lines 152- 157- to better frame research questions there should be a more explicit statement here about elements which are specific to the African context. Elements could include less health funding per capita; more fragmented care due to high proportion of NGO funding; higher background level of communicable disease; PLWMM in Africa are more likely to experience poverty, have little access to a social safety net, and are more likely to experience catastrophic consequences of MM. | Thank you for this valuable suggestion. As described above (results section point a.), we have added a substantial paragraph to contextualize the uniqueness of the multimorbidity challenge in Africa, which is quoted in that section. In spelling this out, we also realized that paragraphs 1 and 2 in the introduction were now largely back to front, and thus we switched the two around making changes to what is now paragraph 1 for clarity/flow. The opening paragraphs pages 7-8 now read:  Multimorbidity – commonly defined as two or more chronic conditions in one person (1) – has become increasingly common as life expectancies rise globally, presenting a profound challenge to organisation of current health systems around single diseases.(2) While the bodies of literature around multimorbidity were initially weighted towards high-income countries (HICs), multimorbidity has more recently been recognised as a global health challenge that may be especially detrimental in low- and middle-income countries (LMICs).(2–4) Priority-setting initiatives for responding to multimorbidity in a global context(3) including in sub-Saharan Africa specifically(5,6) highlight the need to identify common disease ‘clusters’ and their shared determinants; develop integrated prevention and management approaches to jointly address multimorbidity clusters; and to more broadly to restructure health systems to become more holistic and person-centred.(5)  With multimorbidity a growing research priority in Africa, the challenges it presents in this setting is becoming clearer. Like many intractable health system challenges, multimorbidity can be linked to centuries of colonialisation and racial discrimination that have systematically limited access to educational opportunities, employment, adequate housing, and basic healthcare.(7,8) Chronic infectious diseases including HIV and tuberculosis (TB) that have thrived in such contexts increasingly intersect with non-communicable diseases (NCDs) including diabetes, cardiovascular diseases, and mental illness which have sharply risen due to the expansion of HIV treatment coverage, rising life expectancies, and lifestyles of poverty and urbanisation.(7,9,10) The resulting multimorbidity burden has been characterised in terms of the ‘colliding epidemics’ of chronic infectious and NCDs, generally affecting a younger, more economically-active demographic than in HICs, socially patterned along the lines of intersectional inequalities.(4,11) Most people living with multimorbidity (PLWMM) rely on public sector care, which generally remains donor-dependent, siloed, and heavily biased towards HIV and TB, with very limited funding available for NCDs.(4,7) There remains a stark disparity in quality of care for people with NCDs and, despite ongoing efforts, limited progress with integrating NCD care with programmes chronic infectious diseases.(12–14) Fragmented, unevenly-resourced systems place a heavy health, social, and economic on burden on PLWMM, families, and wider social networks, undermining abilities to self-manage conditions and heightening the risk of complications and further morbidities.(15,16).  Just as understandings of multimorbidity in Africa are advancing, it is also becoming clearer that multimorbidity means different things to different stakeholder groups.” |
| • Line 182-188- please include one to two additional sentences on the current controversies with existing MM definitions and what alternative definitions have been suggested. | Thank you, we have added additional sentences and references to highlight these controversies. The sentences on page 8 now read:  “Debates remain as to how many and which conditions ‘count’ as multimorbidity;(16,17) whether it should be expanded to include causes and consequences as well as presence/absence;(6) and indeed, whether the very concept reflects a culture of super-specialised biomedicine in the global North that may not translate well within low-resource settings.(7,9,18)” |
| Methods  • Line 212- further water down claims to following grounded theory methodology as authors did not use intersperse analysis and theoretical sampling but used thematic analysis. I suggest the methodological claim could be re-stated as “This was a qualitative study influenced by principles of participatory research and constructivist grounded theory methodology.” I feel inclusion of the word ‘participatory’ is important because participants contributed to analysis and dissemination. | Thank you for the generous suggestion for rewording. We have combined your suggestion with some of the original words to slightly water down the claims re. grounded theory. The line on page 9 now reads: “We used an inductive, co-productive research design influenced by principles of participatory research and constructivist grounded theory” |
| • Line 225- Text should match diagram, particularly related to what disciplines of clinicians were present (i.e., nursing and physicians). Also missing here is that civil society and community representatives were present. Additional details on what kind of community and civil society representation was present would be helpful. | Thank you, we have additional text to clarify the different disciplines in alignment with the diagram, and clarified the community representation on page 10.  “The collaborator group drew together 60 researchers, clinicians, health planners, and policymakers (HIC-based n=19, LMIC-based (n=41), together representing a wide range of disciplinary perspectives, including from (sub-)fields of epidemiology, public health, clinical medicine, the social sciences and community advocacy (Figure 1).” |
| • Line 259- If it is true that the data in this article limited to the first thematic domain only (i.e., framings and concepts of MM) ,then this should be clearly stated. | Thank you, we found that while the first theme was most explicitly about the framing of multimorbidity, concepts and framings were discussed throughout the workshop, and hence the whole workshop was included for analysis. |
| • There needs to be a distinct data collection section. The sentences at lines 281- 288 should be moved to the data collection section. Also, information is needed on how the data collectors were instructed to take notes, how detailed the notes were (e.g., were comments summarized by speaker or summarized for the whole group?, how were notes anonymized?, how were notes collated and disseminated amongst participants?) | Thank you, we have opened up a discrete data collection section and moved the suggested lines into this section. We have further added information about how the rapporteurs were instructed to take notes, how notes were collated, shared, and anonymized before being deposited. The section pages 12-13 now reads:  *“Data collection*  Source material collected during the workshop included, most centrally, detailed notes of proceedings, including both focus groups and plenary discussions, taken by the rapporteur team. Rapporteurs were instructed to capture summaries of what speakers had said and, where points were made that the rapporteurs felt were especially significant, direct quotes were also taken. Where necessary, the rapporteurs went back to individuals for clarification on points made, which were absorbed into their notes. Also absorbed into the final account were the flip chart pages composed during focus groups and the Microsoft Word documents produced using a shared screen during plenary discussion. Finally, all source materials were reconciled to produce a unified account of both focus-group and plenary discussions. These were made available to all in unanonymised form through a secure online storage platform, before being anonymised in the final deposited dataset.” |
| • Line 292- 299- Could these lines be rephrased to be more explicit and chronological? (For example, first the coding team reviewed all/some of the notes, then there was a meeting amongst the coding team when the initial framework was developed, then XX and YY applied the coding framework to all/some of the notes, had another meeting…) | Thank you, we have made the analysis process more explicit and chronological. The lines on page 13 now read:  “After the rapporteurs had produced the final account of proceedings, the joint first authors (JD, BM, MJN, AS, IGS, SS, MVP) subsequently conducted iterative inductive thematic analysis on the data using a team-based, ‘open coding’ approach that is designed to optimise inter-coder consensus in the context of collaborative research (27) To facilitate this process, data were transferred onto a shared drive, which enabled the coding team to code collaboratively in real time. After an initial pass through the data, the team met to consolidate initial codes into a provisional framework, which was subsequently applied to the whole dataset by JD and MP. A second meeting was used to further refine the framework, with an emphasis on agreeing higher-level themes that grouped and explained lower-level ones. The final framework, the main themes from which are presented in the results section, was then taken to the larger working group and used to develop of an expanded model of multimorbidity. Developing and refining this model was itself an iterative process, involving the working group assembling a tentative visual model based on the coding framework and relevant literature, which was circulated by email to the wider collaborator group along with the draft manuscript. Feedback was used to refine the model before being sent back for a final round of comment and refinement.” |
| o Line 292 - Were initial themes identified or initial codes identified? | Thank you, we meant codes – we have amended the word from “themes” to “codes” (page 13) |
| O Line 295 – Is the final code tree the three themes? I don’t see a coding tree in the results section. | Thank you, we have clarified that the themes and sub-themes presented at the start of the results are those most relevant to the conceptualization of MM (rather than the codes and themes which were determined to not be directly relevant or usefully presented within this manuscript i.e. went beyond the topic of conceptualizing MM, e.g. health system implications of multimorbidity). |
| O Line 295-299- I assume this is the process by which the model in the discussion section was developed, but this process could be more clearly described. (For example, first XX and YY consulted the relevant literature, then developed a tentative visual model based on literature and data. The draft model was then circulated by email to all participants, received feedback—revised the model, repeated the process...) | Thank you, yes this is correct. We have revised these lines on page 13 accordingly, which now read:  “The final framework, which we present at the start of the results section, was then taken to the larger working group and used to develop of an expanded model of multimorbidity. Developing and refining this model was itself an iterative process, involving the working group assembling a tentative visual model based on the coding framework and relevant literature, which was circulated by email to the wider collaborator group along with the draft manuscript. Feedback was used to refine the model before being sent back for a final round of comment and refinement" |
| Results  • Line 317- Consider choosing a different word rather than emerge. Themes are produced (developed, generated, created…) by researchers based on data. | Thank you, we have adjusted to use constructivist vocabulary in discussing the production of themes |
| • Line 370- Can you explain what is meant by secondary complications? Do you mean biomedical (e.g., diabetic nephropathy) or social complications (e.g., loss of employment and housing)? | Thank you, in this context we mean secondary biomedical complications, and have made this clearer in text, page 17:  “First, a concern voiced particularly by clinicians was that it often matters far less to people what the diagnoses and their causes are than their consequences and impacts, which become especially complex when it comes to secondary medical complications (e.g.. diabetic nephropathy)”. |
| Discussion  • Line 495 – Again, reconsider use of the word “revealed”, choose instead generated, developed, created, etc. | As above, we have amended to use constructivist vocabulary to describe the analysis process, though in the process of other revisions requested to the analysis section this specific issues has been resolved anyway. |
| Overall writing  • Typos on line 160, 603, 320 | We have now corrected these; thank you for pointing these errors out |
| • Missing references on line 563 | Thank you, these have been added |
| Abstract  • Revise to suit changes to text (e.g., using more plain language to describe themes, revising methodology claims, include more detail on analysis and data collection | Thank you, we have amended the abstract to include the further requested information. It now reads:  “Multimorbidity is an emerging challenge for health systems globally. It is commonly defined as the co-occurrence of two or more chronic conditions in one person, but its meaning remains a lively area of academic debate, and the utility of the concept beyond high-income settings is uncertain. This article presents the findings from an interdisciplinary research initiative that drew together 60 academic and applied partners working in 10 African countries to answer the questions: how useful is the concept of multimorbidity within Africa? Can the concept be adapted to context to optimise its transformative potentials? During a three-day concept-building workshop, we investigated how the definition of multimorbidity was understood across diverse disciplinary and regional perspectives, evaluated the utility and limitations of existing concepts and definitions, and considered how to build a more context-sensitive, cross-cutting description of multimorbidity. This iterative process was guided by the principles of grounded theory and involved focus- and whole-group discussions during the workshop, thematic coding of workshop discussions, and further post-workshop development and refinement. Three thematic domains emerged from workshop discussions: the current focus of multimorbidity on constituent diseases; the potential for revised concepts to centre the priorities, needs, and social context of people living with multimorbidity (PLWMM); and the need for revised concepts to respond to varied conceptual priorities amongst stakeholders. These themes fed into the development of an expanded conceptual model that centres the catastrophic impacts multimorbidity can have for PLWMM, families and support structures, service providers, and health systems.” |
